# Supplementary material for: Melanocytes and photosensory organs share a common ancestry that illuminates the origins of the neural crest
Source: Commun Biol. 2025 Jul 23;8:1092. doi: 10.1038/s42003-025-08502-0 (PMC12284045; doi:10.1038/s42003-025-08502-0)
Supplement: Supplementary file 1 — All Supplementary Figures with captions in a single file [file 42003_2025_8502_MOESM1_ESM.pdf]

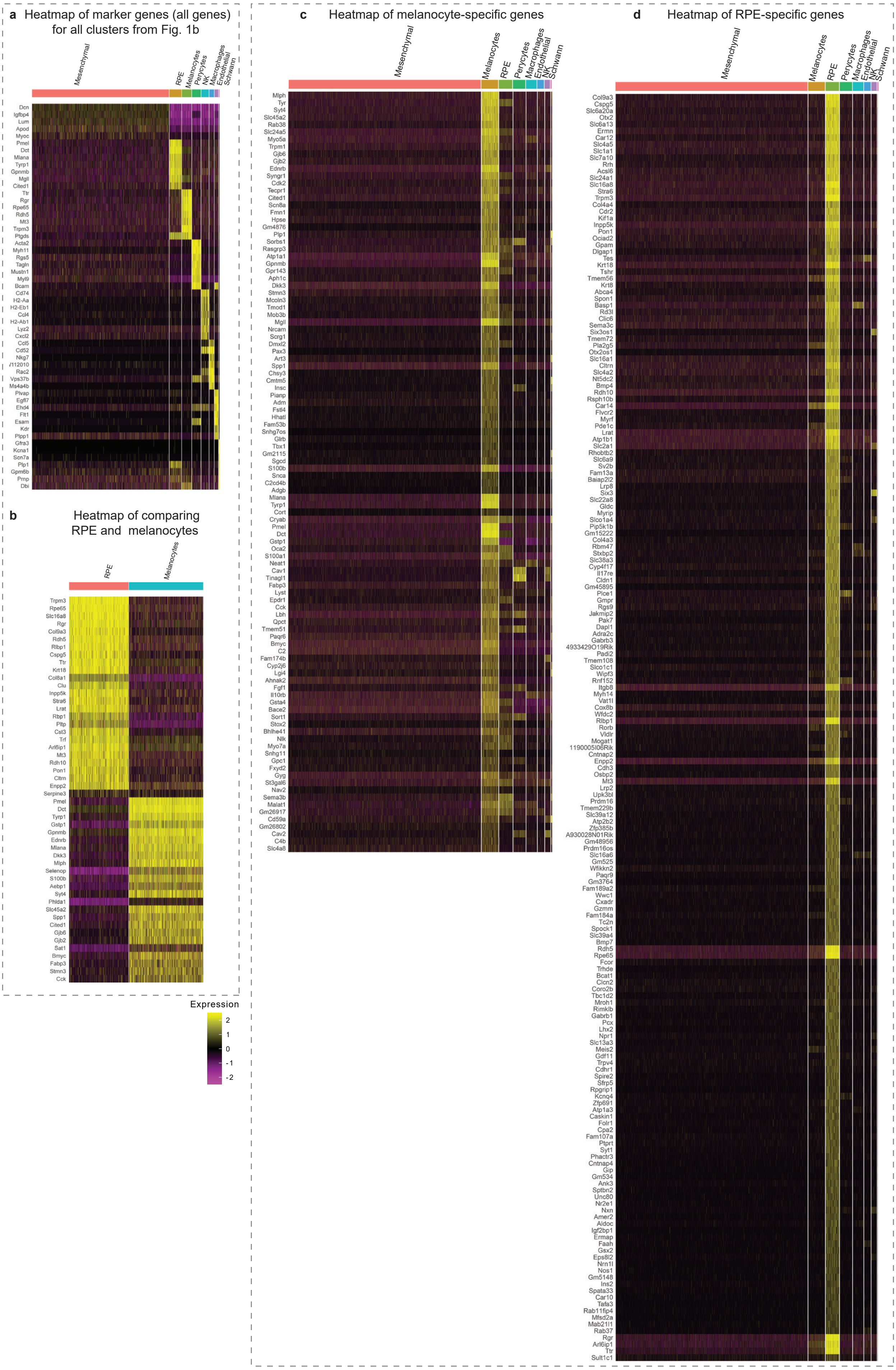

Supplementary Figure 1. Heatmaps of marker genes for clustering in Fig. 1b. a, Marker genes for all identified clusters in Fig 1b**. b,** Differential gene expression distinguishing RPE and melanocytes **c-d,** Heatmap showing the extended lists of melanocyte-enriched (**c**) and RPE-enriched (**d**) expressed genes from Fig. 1b. Heatmaps reflect log-normalized gene expression values.

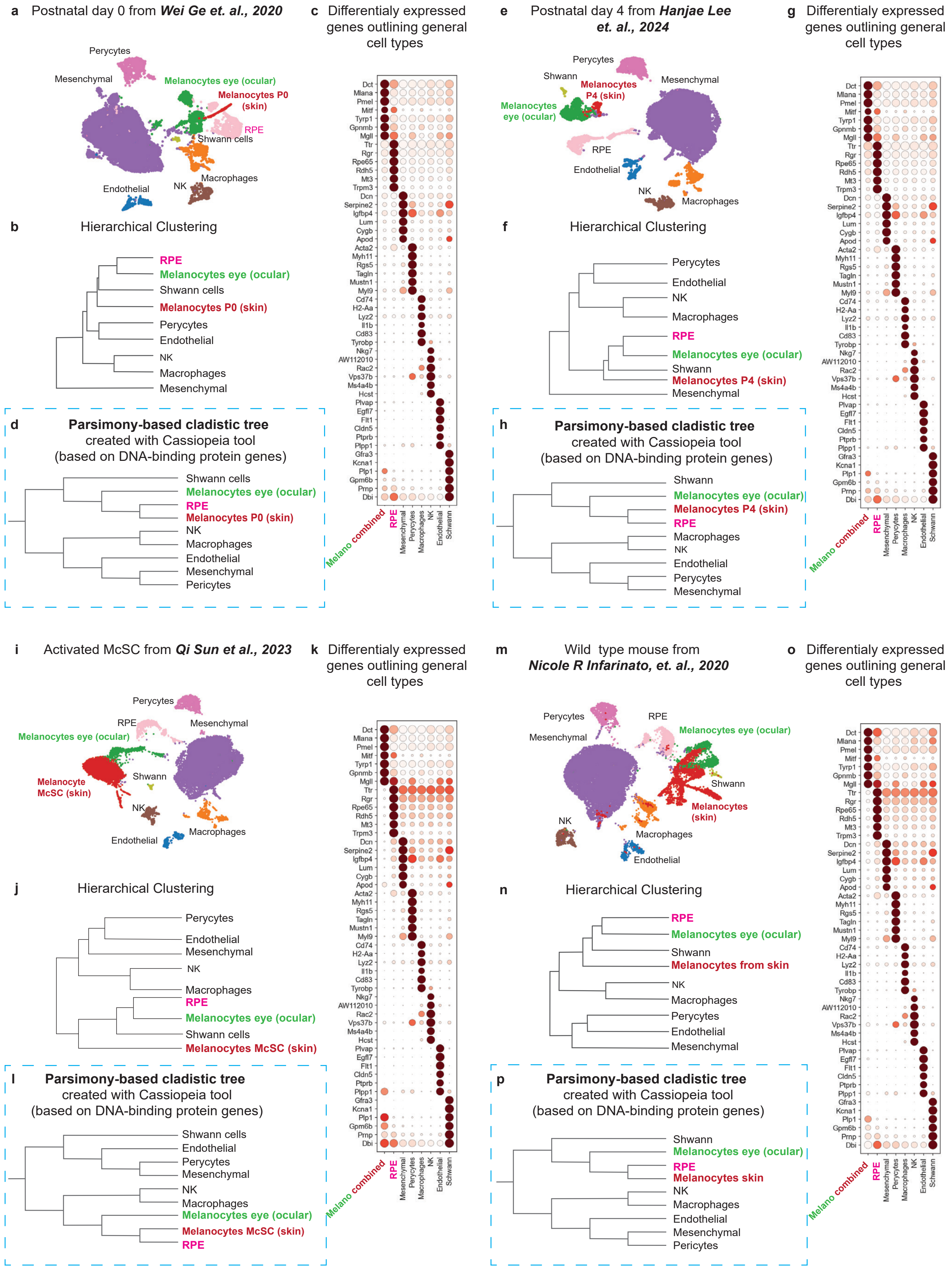

Supplementary Figure 2. Anterior eye and skin-derived melanocytes show robust signatures shared with RPE independently of melanocyte spatial origin. a, UMAP plot showing integration of the anterior eye chamber cell types (Hyungwoo Lee et al., 2022) with skin melanocytes from postnatal day 0 (Wei Ge et al., 2020), annotated by major cell types. **b,** Hierarchical clustering of the integrated cell populations showing close relationships among RPE, Schwann cells, ocular melanocytes, and skin melanocytes. **c,** Dot plot showing general markers for the clusters. **d,** Parsimony-based cladistic dendrogram generated with the Cassiopea tool, illustrating lineage relationships and confirming the close proximity of skin-derived melanocytes to RPE cells. **e–h,** The same analysis using skin melanocytes from postnatal day 4 (Hanjae Lee et al., 2024). **i–l,** The same analysis using skin activated melanocyte stem cells (Qi Sun et al., 2023). **m–p,** The same analysis using skin wild-type skin melanocytes (Nicole R. Infarinato et al., 2020), consistently showing shared clustering and lineage proximity between skin melanocytes and RPE cells across all datasets.

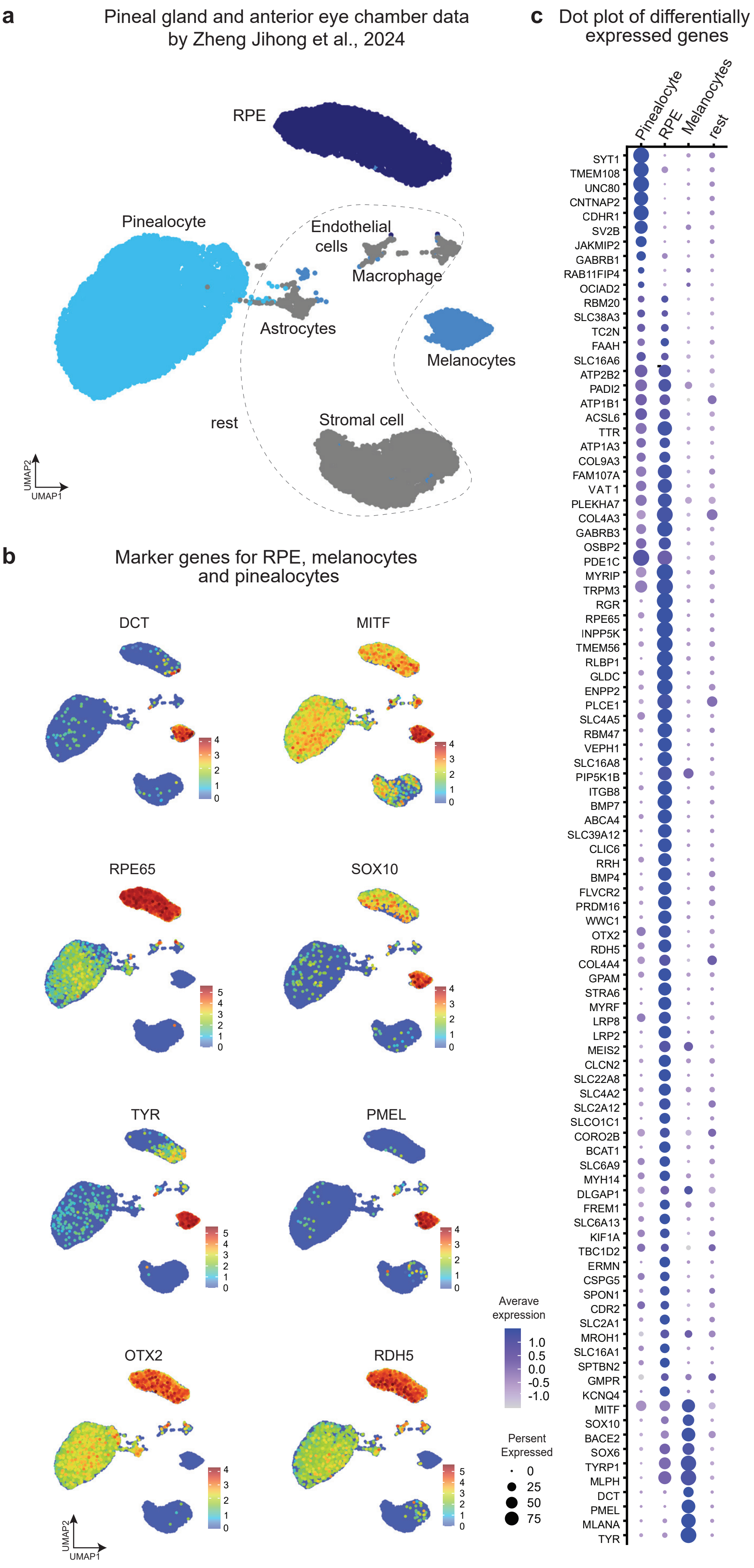

Supplementary Figure 3. Pinealocytes show traces of pigmentation program and reveal similarities with RPE and melanocytes. a, Co-clustering and re-analysis of murine pinealocytes and cells inhabiting the anterior eye chamber (including melanocytes and RPE) from Zheng et al., 2024. **b,** Expression of melanocyte- and RPE-enriched marker genes in pinealocytes. **c,** Dot plot showing the expression of melanocyte- and RPE-enriched genes across all cell types shown in panel a.

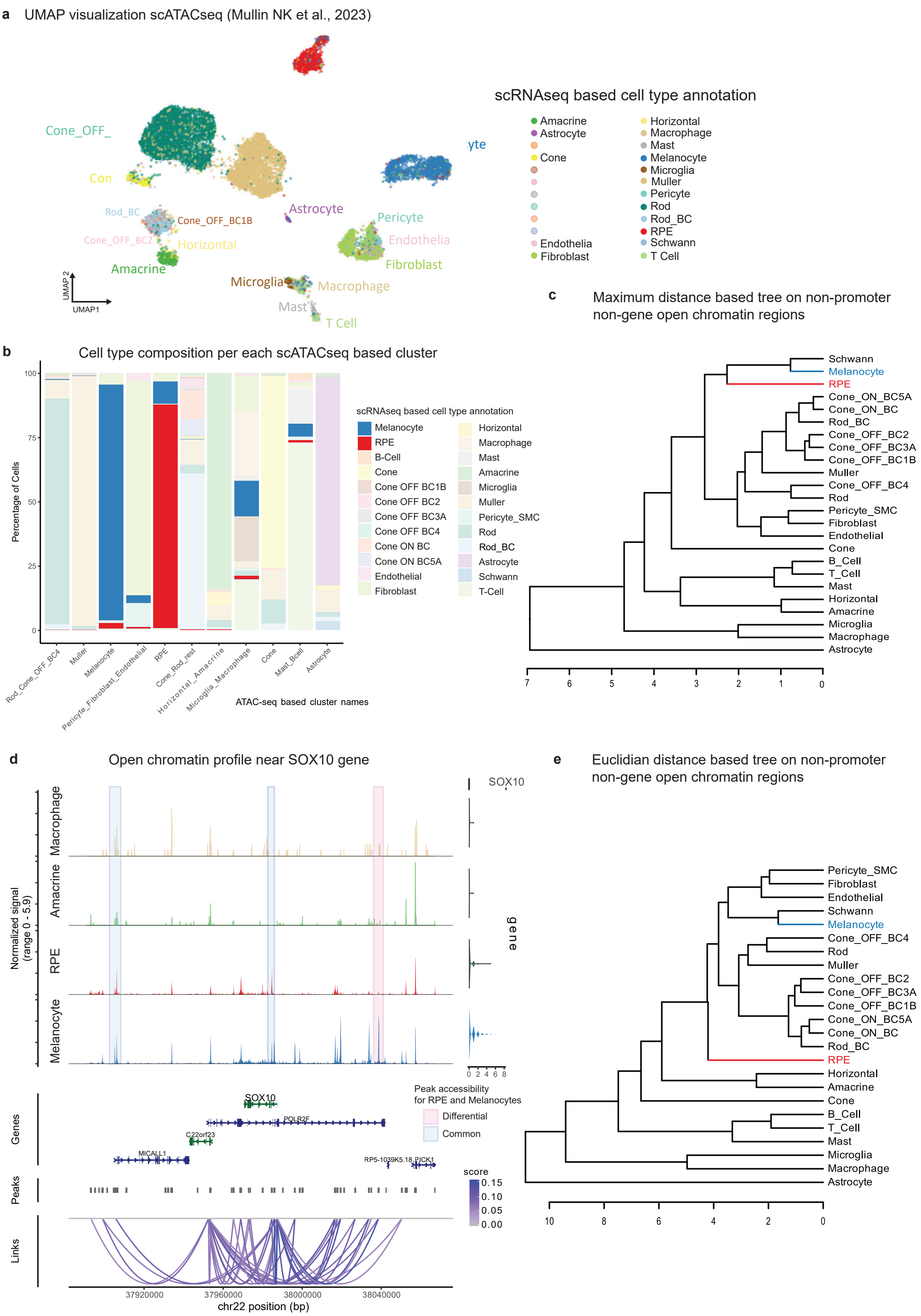

Supplementary Figure 4. Multiome P1-Chromatin accessibility data supports common lines of regulation between RPE and Melanocytes. **a**, Overview of multimodal single-cell RNAseq and ATACseq dataset of human donor eye (from Mullin NK *et al*, 2023), consisting of retinal and choroid tissues. UMAP embedding of cells based on scATACseq part of the data; cell types are annotated based on original scRNAseq data from the same study. **b**, Cell type composition per each scATAC-seq-based cluster. X-axis shows names of clusters based on the majority of scRNAseq-annotated cell types composing the cluster in the scATACseq analysis: for this reason, clusters can have merged names, for example, Pericyte_Fibroblast_Endothelial. Y-axis shows percentage of each scRNAseq-based cell type in those ATAC-seq-based clusters. **c**, Maximum distance-based dendrogram of cell types built with scATACseq data. Promoter regions (2000 bp. upstream and 2000 bp. downstream of TSS) and open chromatin regions within the genes were excluded from scATACseq data prior to calculating the distances between cell type centroids based on LSI dimensionality reduction of scATACseq data. d, Open chromatin profile near SOX10 gene shows peak similarity (blue windows), as well as differences (red windows) in RPE and melanocytes. X-axis indicates genomic coordinates, genes, open chromatin peaks and correlation links between peaks and gene expression. "Links panel" located above shows links correlation coefficient (colored by the correlation score-lower right bar) between the gene expression and the accessibility of the peak (showed in the peaks panel). "Genes panel" visualizes genes that are present inside of the visualized chromatin region; Y-axis shows normalized pseudo-bulk ATAC-seq signal per cell type. Panels on the right side show the expression level of Sox10 for visualized cell types; e, Euclidian distance-based tree on non-promoter and non-gene open chromatin regions, filtered the same way as in the panel c.

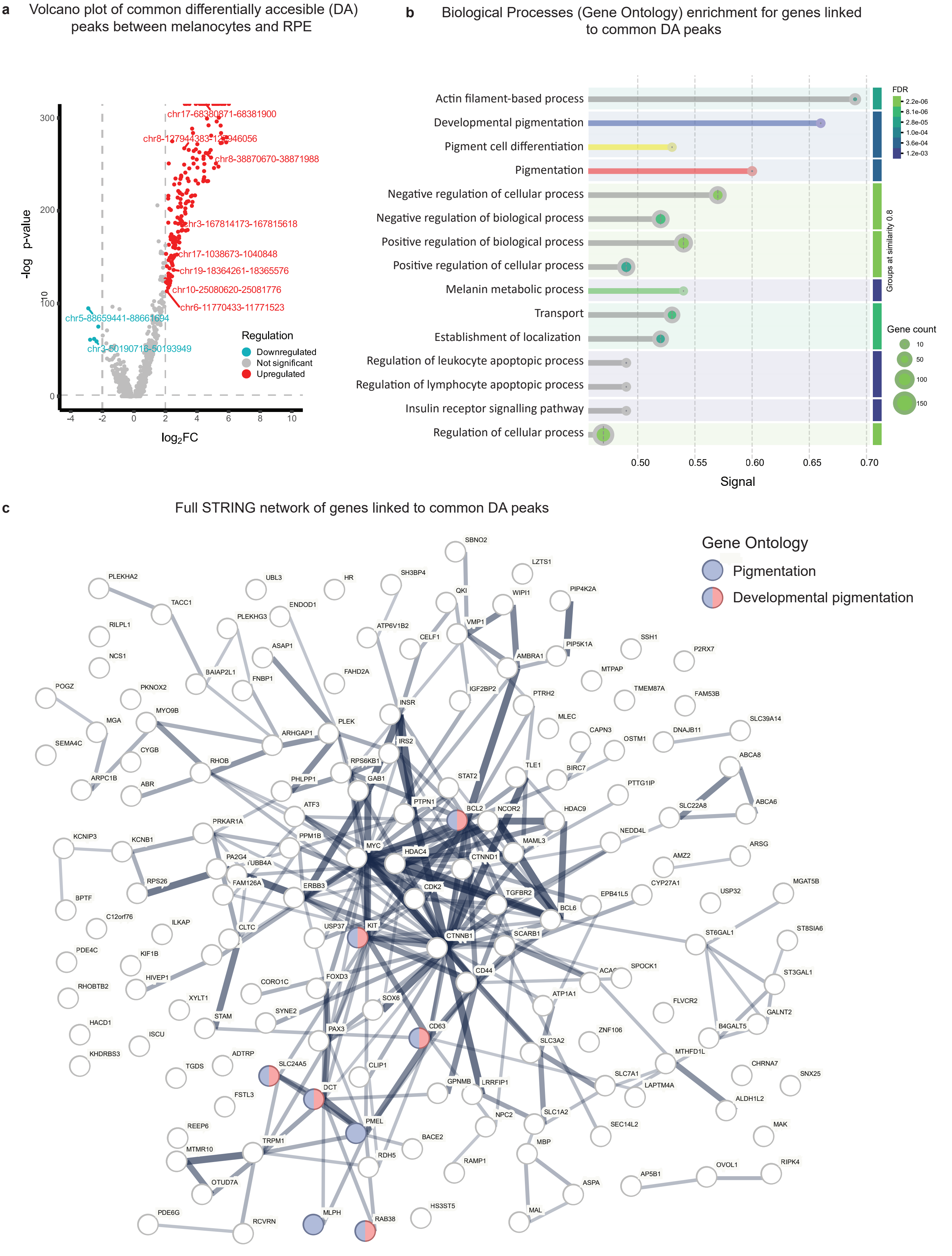

Supplementary Figure 5. Similarly regulated processes in RPE and melanocytes based on comparative analysis of chromatin accessibility. **a**, Volcano plot visualizes differentially accessible (DA) peaks common for melanocytes and RPEs as compared to other cell types. Red dots indicate DA peaks that are upregulated as compared to other cell types, whereas the blue dots show downregulated DA peaks as compared to the rest of cell types. **b,** Enriched biological processes (Gene Ontology) visualized with STRING tool for genes linked to DA peaks. **c,** The network of interacting genes/proteins linked to DA peaks. Nodes show proteins, with blue and red highlighting those related to pigmentation and development of pigmentation. Network edges indicate interaction evidence, where thickness corresponds to the strength of the cumulative supporting data from STRING database.

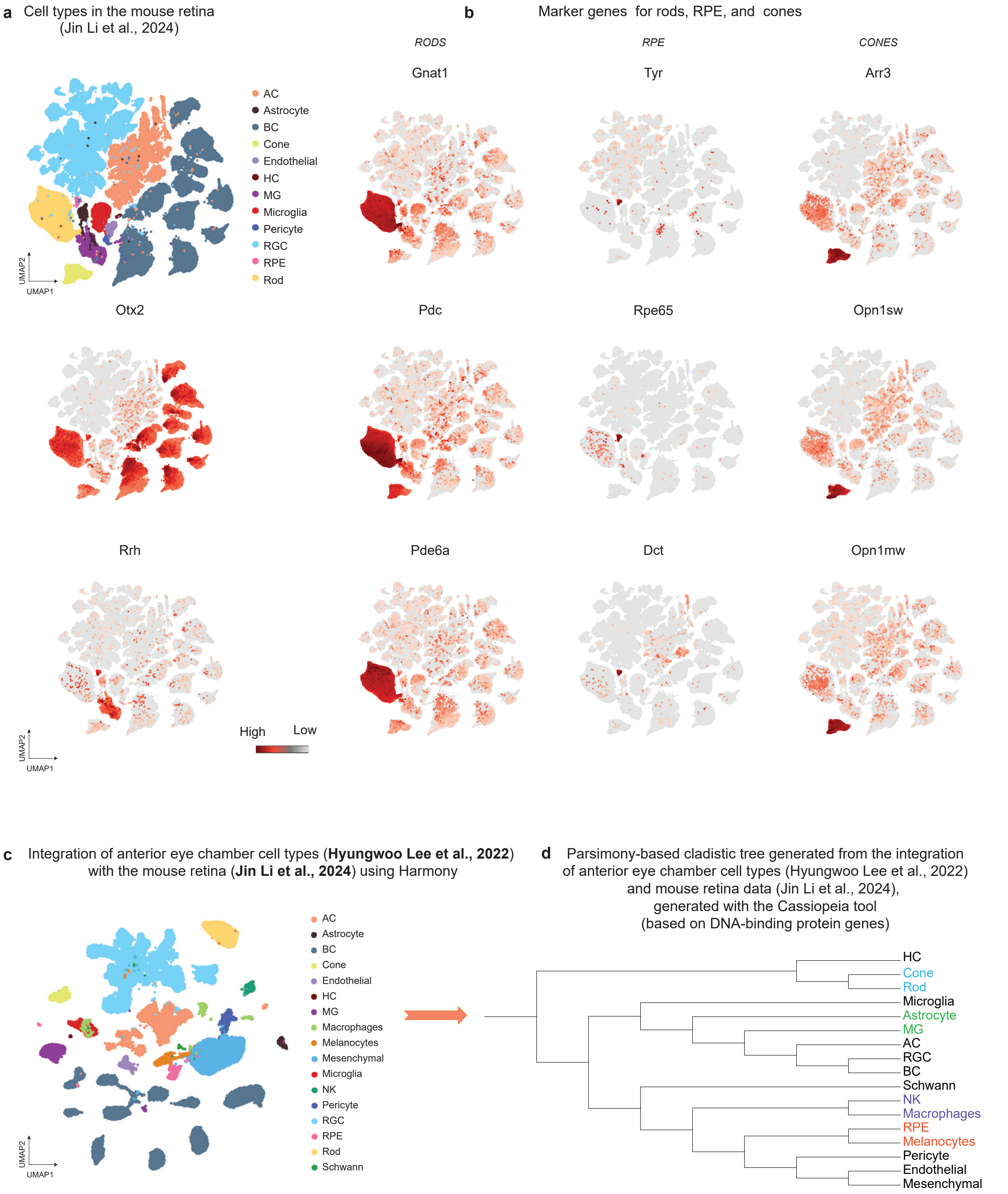

Supplementary Figure 6. Cladistics analysis of the extended set of ocular cell types including melanocytes and RPE (based on the DNA-binding protein code and CASSIOPEIA tool). a, UMAP embedding of mouse retina single-cell transcriptomic data (Li et al., 2024), including clusters for AC (amacrine cells), astrocytes, BC (bipolar cells), cones, endothelial cells, HC (horizontal cells), MG (Müller glia), microglia, pericytes, RGC (Retinal ganglion cells), RPE and rods. **b,** UMAP showing expression of key marker genes for rods (*Gnat1, Pdc, Rrh, Pde6a*), cones (*Arr3, Opn1mw, Opn1sw*), and RPE (*Tyr, Rpe65, Dct*). **c,** UMAP resulting from integration of mouse retina cell types (Li et al., 2024) with the anterior eye chamber cell types (Lee et al., 2022) using Harmony, showing clustering of melanocytes, RPE, and other cell types (the extended set). **d,** Dendrogram generated with the cladistics part of the CASSIOPEA tool based on DNA-binding proteins, highlighting lineage relationships between cell types and supporting a close connection between RPE and melanocytes. Note that other cell types, for which the evolutionary connections are previously established, are also paired by this dendrogram, including rods and cones (cyan), astrocytes and Müller glia (green), immune cell types (blue).

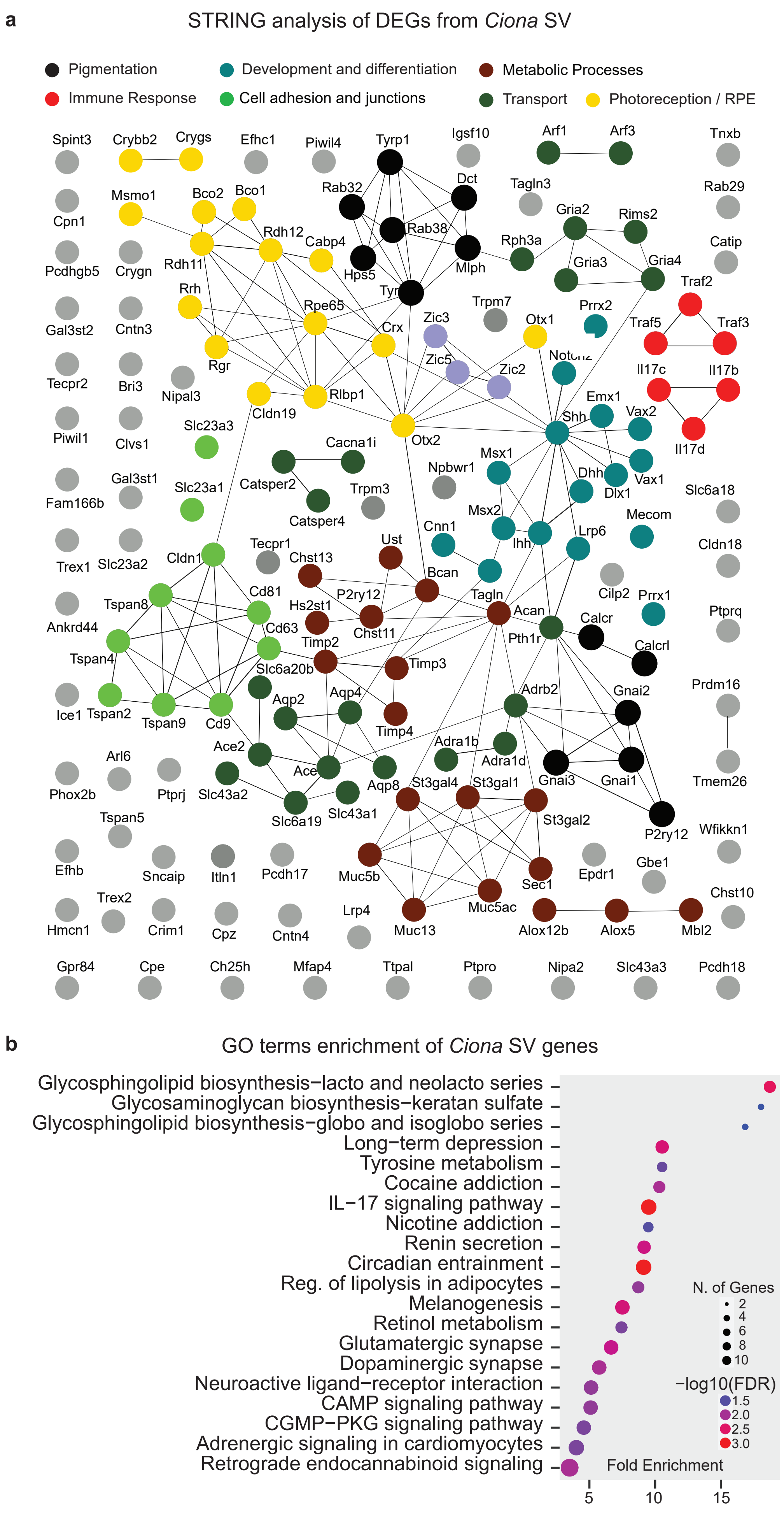

Supplementary Figure 7. a, STRING analysis of interacting protein networks based on genes differentially expressed in *Ciona*'s sensory vesicle. Note the presence of black and yellow subnetworks corresponding to a pigmentation module and a photoreception module correspondingly. **b,** GO-terms enrichment of differentially expressed sensory vesicle genes from *Ciona intestinalis*.
